# Intravoxel incoherent motion MR imaging in nasopharyngeal carcinoma: comparison and correlation with dynamic contrast enhanced MR imaging

**DOI:** 10.18632/oncotarget.19575

**Published:** 2017-07-26

**Authors:** Vincent Lai, Victor Ho Fun Lee, Ka On Lam, Bingsheng Huang, Queenie Chan, Pek Lan Khong

**Affiliations:** ^1^ Department of Diagnostic Radiology, Li Ka Shing Faculty of Medicine, University of Hong Kong, Queen Mary Hospital, Hong Kong, China; ^2^ Department of Clinical Oncology, Li Ka Shing Faculty of Medicine, University of Hong Kong, Queen Mary Hospital, Hong Kong, China; ^3^ Department of Biomedical Engineering, School of Medicine, Shenzhen University, Shenzhen, China; ^4^ Philips Healthcare, Hong Kong, China

**Keywords:** nasopharyngeal carcinoma, intravoxel incoherent motion, dynamic contrast enhanced, magnetic resonance imaging, tumor staging

## Abstract

**Objectives:**

To compare accuracy and assess agreement between intravoxel incoherent motion (IVIM) magnetic resonance (MR) perfusion-related parameters and quantitative dynamic contrast-enhanced (DCE) MR parameters in nasopharyngeal carcinoma (NPC).

**Results:**

*D*, *f*, D*, *K^trans^*, *K_ep_* and *V_p_* were significantly lower in the high stage group while *V_e_* was significantly higher in the high stage group. Optimal cut-off values were: *D*=0.749 × 10^−3^ mm^2^/s; *f*=0.145; D*=100.401 × 10^−3^ mm^2^/s; *K^trans^*=0.571/min; *K_ep_*=0.8196/min; *V_e_*=0.6556 %; *V_p_*=0.0757 %. D* (*p*=0.001), *K^trans^* (*p*<0.001), *V_e_* (*p*=0.014) were all reliable independent predictors for AJCC staging. IVIM-MR perfusion-related (*f*, D*) and DCE-MR (*K^trans^*, *K_ep_*, *V_e_*, *V_p_*) parameters were significantly correlated (*p*<0.001).

**Materials and Methods:**

75 patients with newly diagnosed NPC were prospectively recruited. Diffusion-weighted MR and DCE-MR imaging were performed with respective IVIM (*D*, *f*, D*) and DCE (*K^trans^*, *K_ep_*, *V_e_*, *V_p_*) MR parameters calculated. Patients were stratified into low and high tumor stage groups according to American Joint Committee on Cancer (AJCC) staging for determination of the predictive powers of IVIM-MR and DCE-MR parameters using t–test, ROC curve analyses and multiple logistic regression analysis. Correlation between IVIM-MR perfusion-related and DCE-MR parameters was assessed using Spearman's rank correlation.

**Conclusion:**

IVIM-MR perfusion-related and quantitative DCE-MR parameters were significantly correlated in the assessment of NPC and were both reliable independent predictors in the prediction of AJCC staging. IVIM-MR perfusion imaging can be a potential useful non-invasive perfusion imaging tool for clinical use in the assessment of NPC.

## INTRODUCTION

Noninvasive diagnostic imaging and staging in nasopharyngeal carcinoma (NPC) is crucial in the early diagnosis and treatment planning, especially for patients with high stage disease requiring adjuvant therapy. Magnetic resonance (MR) imaging has been the gold standard and imaging of choice in the detection and delineation of local tumor extent [1–4]. Diffusion-weighted (DW) MR imaging allows additional information on the functional assessment and characterization of the tumor, showing promising results with successful differentiation between different tumor grading, rendering it an imaging biomarker.

Intravoxel incoherent motion (IVIM) MR imaging is one type of DW MR imaging technique receiving much attraction in that it provides with us both the diffusion and perfusion related information without the use of exogenous contrast. We have previously demonstrated that it is feasible in the differentiation between NPC and post-chemoradiation fibrosis [5], as well as in the pre-treatment staging prediction [6], showing high diagnostic accuracy. While the precision of the derived pure diffusion parameter, *D* (reflecting tissue cellularity) has been proven to be consistent with high diagnostic confidence, concern has been raised regarding the accuracy of the derived perfusion related parameters (perfusion fraction, *f* reflecting normal angiogenesis with intact vascular permeability; and pseudodiffusion coefficient, D* reflecting tumoral vascularity) given the limited signal-to-noise ratio (SNR). The clinical utility of dynamic contrast enhanced (DCE) MR perfusion had been established in various tumors [7–10] as well as in NPC [11–13], with its quantitative analysis being found useful in the assessment of tumoral vascularity, microcirculation property and hypoxia status since the contrast kinetics is associated with angiogenesis within tumors. In particular, *K^trans^* (diffusive transport of Gd-DOTA across capillary endothelium/volume transfer) and *V_e_* (extracellular extravascular volume fraction) are frequently used to assess tumor outcomes as well as patient's prognosis [14]. The relationship between IVIM-MR perfusion-related parameters and semi-quantitative DCE-MR parameters had initially been tested in NPC, showing promising result [15]. However, the relationship between IVIM-MR perfusion-related parameters and quantitative DCE-MR parameters remains unexplored.

The aim of this study was to assess the correlation and agreement of the IVIM-MR perfusion-related parameters with the quantitative DCE-MR parameters, and to compare their diagnostic performance.

## RESULTS

The mean tumor volume was 1702.8 mm^3^ +/− 2012.9 mm^3^ (44.4 – 10098.6 mm^3^). The interobserver agreement showed good agreement with the kappa value (κ) for *D, f*, D*, K^trans^, *K_ep_*, *V_e_* and *V_p_* measured at 0.86, 0.77, 0.70, 0.81, 0.76, 0.80 and 0.72 respectively.

### IVIM DW-MR parameters

**Figure 1:**
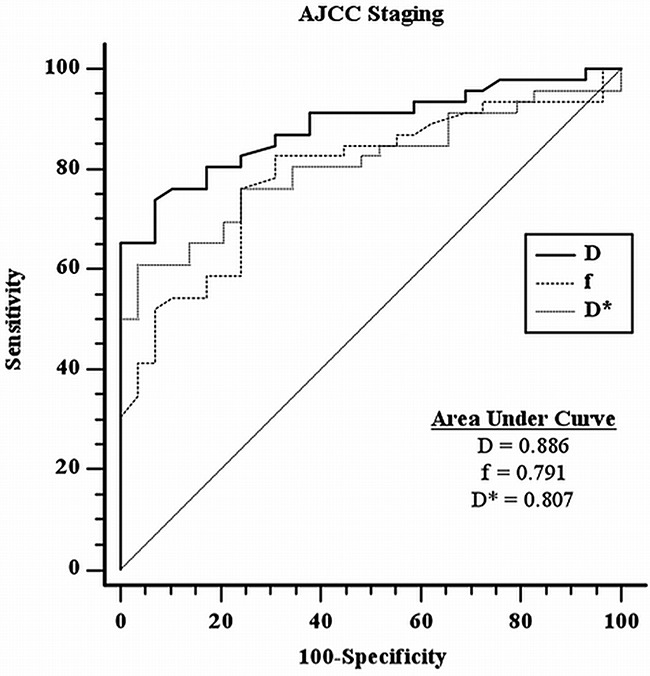
ROC curves analysis for IVIM-MR parameters with respective areas under curves in AJCC staging

Both the diffusion (*D*) and perfusion-related parameters (*f*, D*) were statistically significantly lower in the high stage group as compared with low stage group in AJCC staging. Their respective mean values +/− standard deviation (SD) were summarized in Table 1. Respective optimal cut-off values upon ROC curve analyses (with respective sensitivity, specificity, positive likelihood ratio and negative likelihood ratio) were summarized in Table 2 and were as follows: *D*=0.749 × 10^−3^ mm^2^/s (73.9%, 93.1%, 10.72, 0.28); *f*=0.145 (76.1%, 75.9%, 3.15, 0.32); and D*=100.401 × 10^−3^ mm^2^/s (60.9%, 96.6%, 17.65, 0.41); Respective ROC curves and areas under curves were represented in Figure 1. *D* was the most powerful parameter based on the area under curve (AUC=0.886).

**Table 1:** Summarized mean values of the IVIM-MR parameters (D, f, D*) and DCE-MR parameters (*K^trans^*, *K_e_*_*p*_, *V*_*e*_ and *V*_*p*_) with respective *p* values between low stage and high stage groups for AJCC staging according to Student's t-test analysis

AJCC stage	*n*(75)	*D*(x 10^−3^ mm^2^/s)	*f*	D*(x 10^−3^ mm^2^/s)	*Ktrans*(/min)	*Kep*(/min)	*Ve*(%)	*Vp*(%)
Low (I+II)	29	0.803 +/− 0.188	0.157 +/− 0.096	109.853 +/− 63.651	0.645 +/− 0102	0.885 +/− 0.213	0.555 +/− 0.311	0.088 +/− 0.077
High (III+IV)	46	0.700 +/− 0.193	0.130 +/− 0.114	98.686 +/− 64.372	0.511 +/− 0.137	0.748 +/− 0.227	0.869 +/− 0.631	0.065 +/− 0.069
*t-test*		*p*<0.001	*p*<0.001	*p*<0.001	*p*<0.001	*p*<0.001	*p*<0.001	*p*<0.001

* AJCC = American Joint Committee on Cancer.

**Table 2:** Optimal cut-off values under 95% confidence interval for AJCC staging according to ROC curves analysis, with p values from direct comparison of ROC curves (only positive comparisons are shown)

AJCC stage	Cut off value	Sensitivity(%) *(95% CI)*	Specificity(%) *(95% CI)*	Pos LR*(95% CI)*	Neg LR*(95% CI)*	*p* value
*D*	0.749 × 10^−3^ mm^2^/s	73.9*(58.9-85.7)*	93.1*(77.9-99.2)*	10.72*(2.8-41.3)*	0.28*(0.2-0.5)*	
*f*	0.145	76.1*(61.24-87.4)*	75.9*(56.5-89.7)*	3.15*(1.6-6.1)*	0.32*(0.2-0.5)*	
D*	100.401 × 10^−3^ mm^2^/s	60.9*(45.4-74.9)*	96.6*(82.2-99.9)*	17.65*(2.5-122.8)*	0.41*(0.3-0.6)*	
*K^trans^*	0.571 /min	97.8*(88.5-99.9)*	96.6*(82.2-99.9)*	28.37*(4.1-194.7)*	0.023*(0.003-0.2)*	0.0001^a,b^
*K_ep_*	0.8196 /min	91.3*(79.2-97.6)*	86.2*(68.3-96.1)*	6.62*(2.7-16.5)*	0.10*(0.04-0.3)*	
*V_e_*	0.6556 %	87.0*(73.7-95.1)*	89.7*(72.6-97.8)*	8.41*(2.9-24.7)*	0.15*(0.07-0.3)*	
*V_p_*	0.0757 %	84.8*(71.1-93.7)*	93.1*(77.2-99.2)*	12.29*(3.2-47.1)*	0.16*(0.08-0.3)*	

* AJCC = American Joint Committee on Cancer; CI = confidence interval; Pos LR = positive likelihood ratio; Neg LR = negative likelihood ratio “a” = *K^trans^* vs *f*; “b” = *K^trans^* vs D*;

*Bonferroni corrected p value = 0.002*.

### DCE-MR parameters

**Figure 2:**
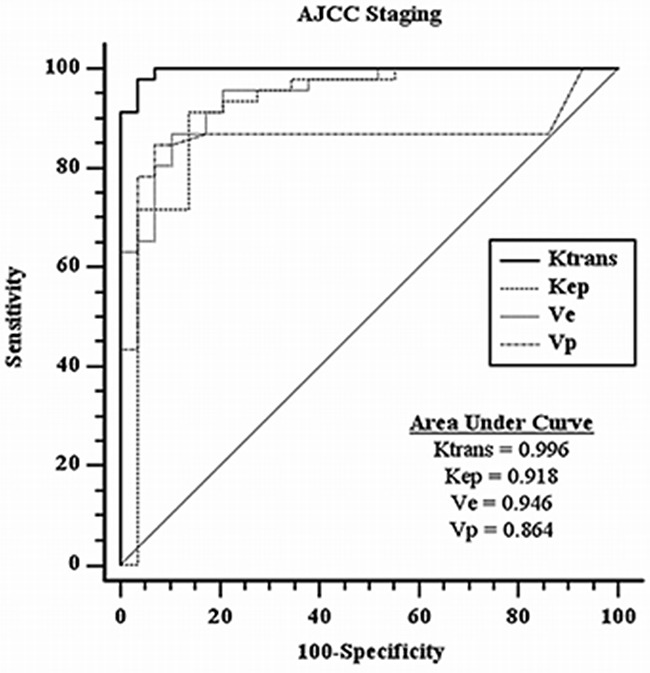
ROC curves analysis for DCE-MR parameters with respective areas under curves in AJCC staging

*K^trans^*, *K_ep_* and *V_p_* were all statistically significantly lower in the high stage group as compared with low stage group in AJCC staging. *V_e_* on the contrary, was found to be positively correlated, showing statistically significantly higher value in the high stage group as compared with low stage group. Their respective mean values +/− SD were summarized in Table 1. Respective optimal cut-off values upon ROC curve analyses (with respective sensitivity, specificity, positive likelihood ratio and negative likelihood ratio) were summarized in Table 2 and were as follows: *K^trans^*=0.571 /min (97.8%, 96.6%, 28.37, 0.023); *K_ep_*=0.8196/min (91.3%, 86.2%, 6.62, 0.10); *V_e_*=0.6556 % (87.0%, 89.7%, 8.41, 0.15); *V_p_*=0.0757 % (84.8%, 93.1%, 12.29, 0.16). Respective ROC curves and areas under curves were represented in Figure 2. *K^trans^* was the most powerful parameter based on AUC (0.996).

### Comparison between IVIM-MR and DCE-MR parameters

Stepwise regression analysis revealed *D* (*p*<0.001), *f* (*p*=0.018), D* (*p*=0.001), *K^trans^* (*p*<0.001) and *V_e_* (*p*=0.014) were all reliable independent predictors. *K^trans^* was statistically significantly more powerful than *f* and D* (*p*=0.001) on direct comparison. No statistical significance could be reached when *V_e_* was compared against *D, f* and D*.

### Correlation between IVIM and DCE-MR parameters

**Figure 3:**
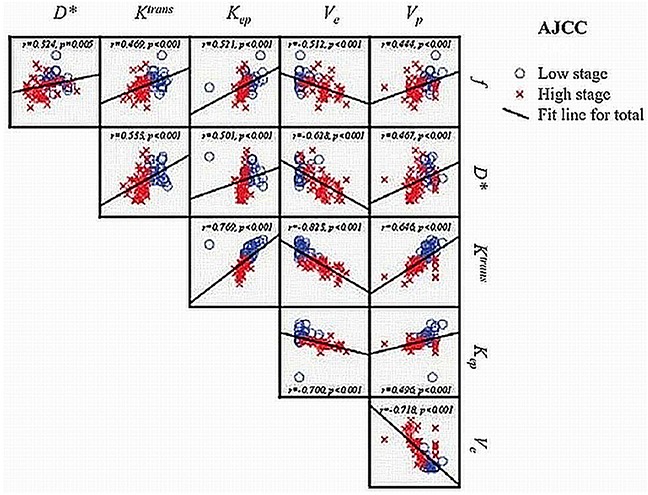
Scatter plots and Spearman rank correlation coefficient with 2-tailed statistical significance (p value) between IVIM-MR parameters and DCE-MR parameters r = Spearman rank correlation coefficient

There was significant correlation between IVIM-MR perfusion-related parameters, *f* and D* with all DCE-MR parameters, *K^trans^*, *K_ep_*, *V_e_* and *V_p_* (Spearman's rank correlation coefficients: −0.628 to 0.533, *p* <0.001 *(Bonferroni corrected p value = 0.002)*). Their results were summarized and represented by scatter pots as shown in Figure 3.

## DISCUSSION

Accurate staging in NPC is imperative for treatment planning as the differences in treatment paradigms and prognosis are based on tumor aggressiveness. This emphasizes the need for a noninvasive imaging tool that can help in the prediction of staging. IVIM MR imaging has been an attractive and promising alternative imaging tool that can simultaneously assess tissue vascularity and cellularity without the need of exogenous contrast. Despite the initial promising result of correlation between IVIM-MR perfusion-related and semi-quantitative DCE-MR parameters, the relationship between IVIM-MR perfusion-related and quantitative DCE-MR parameters remains unclear.

Our results are in concordance with other published data on NPC [11–15], showing similar range, trend and distribution of the parameters across different tumor stages. The improvement in results as compared with our earlier DCE-MR study on NPC [12] is likely attributed to the larger cohort of patients and inclusion of wider spectrum of different tumor stages of NPC. Both the IVIM-MR parameters (*D*, *f* and D*) and DCE-MR parameters (*K^trans^* and *V_e_*) were found to be reliable independent predictors in the prediction of AJCC staging. On direct comparison of their diagnostic accuracy, *K^trans^* was more robust as compared with *f* and D* (*p*=0.001). The robustness of quantitative DCE-MR imaging (in particular for *K^trans^*) is likely attributed to its direct reflection of the tissue physiology based on the concentration of contrast and are more closely linked to perfusion and permeability, hence related to tumor angiogenesis [14]. The exact relationship between IVIM-MR perfusion-related parameters and DCE-MR parameters remains unclear, but is likely to demonstrate close correlation as Le Bihan et al. [16] reasoned that tracer delivery to tissue is dependent on intravascular flow. Our results confirmed that IVIM-MR perfusion-related parameters (*f* and D*) were significantly correlated with the quantitative DCE-MR parameters (*K^trans^*, *K_ep_*, *V_e_* and *V_p_*) (Spearman rank correlation *p*<0.001). This helped to suggest the usefulness of the IVIM-MR perfusion-related parameters in that they are potentially reflecting the microvessel density or contrast kinetics as observed with the quantitative DCE-MR parameters, rendering it a potential useful non-invasive perfusion imaging tool in the assessment of angiogenesis or neovasculature in NPC.

High stage NPC exhibited significantly lower perfusion parameters (*f*, D*, K^trans^, *K_ep_* and *V_p_*) as compared with low stage NPC, consistent with earlier IVIM-MR findings [5–6]. This can be attributed to the increasing tumoral heterogeneity due to the presence of microscopic necrosis or hypoxia in association with high stage NPC, as supported by prior studies showing reduced *K^trans^* with increasing hypoxia [17]. Sumi and Nakamura [18] also suggested that different stages of tumors would have distinctive stromal components with varying degrees of vascularization, and hence perfusion level, reflecting the relative area of stromal tissues with varying levels of vessel attenuation. Low stage NPC is less aggressive and has tightly packed stromal tissue due to the absence of necrosis, therefore increased vascularity overall. Our finding of higher *V_e_* in high stage NPC suggests tumor aggression due to larger extracellular volume fraction. Although *V_e_* reflects the extracellular extravascular space but not the vascular compartment, it had been found to correlate with tumor aggressiveness and played a role in stimulating angiogenesis as well as strengthening tumor cells migration into peripheral tissue [11, 14, 17]. Chin et al. [14] also hypothesized that tumor microenvironment with a large *V_e_* would promote metastatic dissemination as it reflected the presence of abundant vascularized extracellular space.

The major limitation of our study is the lack of histopathological correlation such that the physiological basis of the perfusion status between different stages of NPC cannot be established. In particular, *K^trans^* does not necessarily linearly correlated to perfusion as it is also determined by the permeability of the blood vessels. Measurement reliability of both the IVIM-MR and DCE-MR perfusion parameters is another major challenge, given the intrinsic wide variability and SNR variation as reflected by the large SD and heterogeneous parametric maps accompanying *f*, D*, *V_e_* and *V_p_* [19, 20]. The extended Tofts model used in the analysis of DCE-MR perfusion parameters is technically challenging, which can be confounded by many factors in both the acquisition and post-processing stages. In particular, the extremely low *V_p_* value raises potential concern of over-fitting and doubtfulness of its clinical use. In addition, it may not be able to sufficiently integrate the intricate microenvironment of biological tissues [13]. Estimations of IVIM-MR parameters are known to depend upon *b*-value selection as different *b* values can result in higher or lower parameter estimations [21, 22]. The 13 *b*-values utilized in our study were based on our initial experience in our earlier studies, achieving reasonable results. We believe the use of optimal *b*-values distribution and signal averaging using ROI techniques have helped in achieving satisfactory results. The major drawback was the long duration of scanning time (12 minutes) of the DW sequence rendering it undesirable for clinical use. Future study can be directed on different combinations of *b*-values as to search for an optimal number or range for clinical utilization within a reasonable scanning time. Although we were able to demonstrate the feasibility and correlation of IVIM–MR perfusion related parameters, larger sample study is necessary to assess and confirm the diagnostic performance. Larger cohort from different tumor stages of NPC wound also be helpful to look for its discriminating power in individual tumor stages, in particular within same T stage.

In conclusion, we have demonstrated significant correlation between IVIM-MR perfusion-related parameters (*f* and D*) and quantitative DCE-MR parameters (*K^trans^*, *K_ep_*, *V_e_* and *V_p_*) in the assessment of NPC. *D*, *f*, D*, *K^trans^* and *V_e_* were all found to be reliable independent predictors in the prediction of AJCC staging though *K^trans^* was more robust as compared with *f* and D* (*p*=0.001). IVIM-MR perfusion imaging can be a potential useful non-invasive perfusion imaging tool for clinical use in the assessment of NPC.

## MATERIALS AND METHODS

### Patient selection

A prospective study was carried out after institutional review board approval with written informed consent was obtained. Consecutive patients with newly diagnosed NPC seen in our institution from January 2012 to March 2014 were recruited. Major inclusion criterion was patients with newly diagnosed biopsied proven NPC. Major exclusion criteria were: a) treatment of any form (surgery, chemoradiation) already given; b) concurrent nasopharyngeal disease or other tumors.

Eighty patients with newly diagnosed biopsied proven undifferentiated NPC were initially recruited. No other histological subtypes of NPC were identified. Five patients were excluded due to small tumor volumes (<30.00 mm^3^) or significant motion artifacts leading to inaccurate assessment. Therefore, a total of seventy-five patients (51 male, 24 female; mean age 53.0 +/− 13.4 years, range 25-90 years) were included. American Joint Committee on Cancer (AJCC) and TNM stages for all patients were determined by two independent clinical oncologists subspecialized in NPC, based on all available imaging findings and endoscopic findings. Discrepancy if any, was resolved after subsequent consensus. Distribution of all patients’ respective AJCC and TNM stages together with their clinical demographic data were listed in Table 3.

**Table 3:** Summarized clinical and demographic data for all patients

N	75
Sex:	
Male	51
Female	24
Age (years)	53.0 ± 13.4 (25 - 90)
Tumor volume (mm^3^)	1702.8 ± 2012.9 (44.4 – 10098.6)
AJCC staging	
I	7 (9%)
II	22 (29%)
III	29 (39%)
IV	17 (23%)
T staging	
T1	33 (44%)
T2	15 (20%)
T3	16 (21%)
T4	11 (15%)
N staging	
N0	10 (13%)
N1	28 (37%)
N2	33 (44%)
N3	4 (6%)
M staging	
M0	72 (96%)
M1	3 (4%)

* AJCC = American Joint Committee on Cancer.

### MR imaging techniques

MR imaging were performed with a 3.0-T MR scanner (Achieva 3.0T, Philips Healthcare, Best, The Netherlands) utilizing a 16-channel neurovascular coil. Standard conventional sequences (axial T1-weighted turbo spin echo (TSE), axial T2-weighted short T1 inversion recovery (STIR), coronal T2-weighted STIR, 3D T1-weighted turbo-field-echo (TFE) post-contrast scan) were initially performed.

DW MR imaging was subsequently performed using a fat-suppressed single-shot spin-echo echo-planar imaging sequence with the following parameters: TR/TE=7996/43 ms; echo-planar imaging factor=35; sensitivity encoding factor=3.5; FOV=230 × 230 mm; slice thickness=3 mm; intersection gap=0.3 mm; matrix=128 × 128; receiver bandwidth=2735.7 Hz per pixel; motion probing gradients in three orthogonal axes; number of signal averages=3; parallel imaging (SENSitivity Encoding [SENSE]) factor=3. A total of 13 *b*-values were used: 0, 10, 20, 30, 40, 60, 100, 120, 160, 200, 300, 500 and 1000 s/mm^2^. The scanning time was about 12 minutes.

It was then followed by dynamic contrast enhanced MR imaging utilizing a 3D T1-weighted fast-field-echo (FFE) sequence with 2 different flip angles [TR/TE=4.8/2.4 ms; flip angle=5^0^ for precontrast scan & 15^0^ for dynamic scan; FOV=230 × 230 mm; matrix=144 × 144; 65 dynamic acquisitions; number of signal averages=4]. Intravenous bolus injection of 0.1 mmol/kg of body weight Gd-DOTA (Dotarem, Guerbet, France) was administered at the 8^th^ dynamic acquisition at a rate of 3.5 mL/s by power injector followed by 25-mL saline flush. The scanning time was about 10 minutes.

### Image analysis

Image analysis of the DW MRI data was performed using an in-house software program developed in IDL 6.3 (ITT Visual Information Solutions, Boulder, CO) and fitted on a pixel-by-pixel basis by Levenberg-Marquardt algorithm, as described before [23–26]. DW images from all 3 directions were automatically averaged and used for analysis. The bi-exponential model from an IVIM sequence was described by Le Bihan [27] as *S_b_*/*S_0_*= (1-*f*)exp(−*bD*)+*f*exp[−*b*(D*+D)], where *S_b_* is the signal intensity in the pixel with diffusion gradient *b*, *S_0_* is the signal intensity in the pixel without diffusion gradient, *D* is the true diffusion as reflected by pure molecular diffusion, *f* is the fractional perfusion related to microcirculation, and D* is the pseudodiffusion coefficient representing perfusion-related diffusion or incoherent microcirculation. *D* was obtained by a simplified linear fit equation (S_*b*_ = S_0_ x exp-*^b.D^*) using *b* values > 200 s/mm^2^; while *f* and D* were calculated by a nonlinear regression algorithm using all *b* values. Using Matlab (The MathWorks, Natick, Massachusetts), only pixels with signals safely above background noises were included for calculation to avoid fitting pixels with low SNR. Therefore, pixels with low SNR were removed from subsequent analysis.

Image analysis of the DCE MRI data was performed by in-house software program (DCE-Tool, Version 5.1, Philips Healthcare, Best, The Netherlands) based on a two-compartment model (Extend Tofts model) [28, 29]. Motion correction and co-registration were initially performed and both the precontrast T1 map and dynamic T1 map were calculated as described previously [30, 31]. Based on the assumption of a linear relation between Gd-DOTA concentration and 1/T1, the Gd-DOTA concentration map was calculated. Arterial input function (AIF) was selected and obtained from the vertebral artery, with the parametric maps of *K^trans^* (diffusive transport of Gd-DOTA across capillary endothelium/volume transfer), *K_ep_* (rate constant of Gd-DOTA/reflux rate constant), *V_e_* (extracellular extravascular volume fraction) and *V_p_* (intravascular plasma volume fraction) generated automatically. Again, Using Matlab (The MathWorks, Natick, Massachusetts), only pixels with signals safely above background noises were included for calculation to avoid fitting pixels with low SNR. Any unphysiological data such as zero were excluded. Kinetic pharmacokinetic model fitting curves were used for validation of the data.

**Figure 4:**
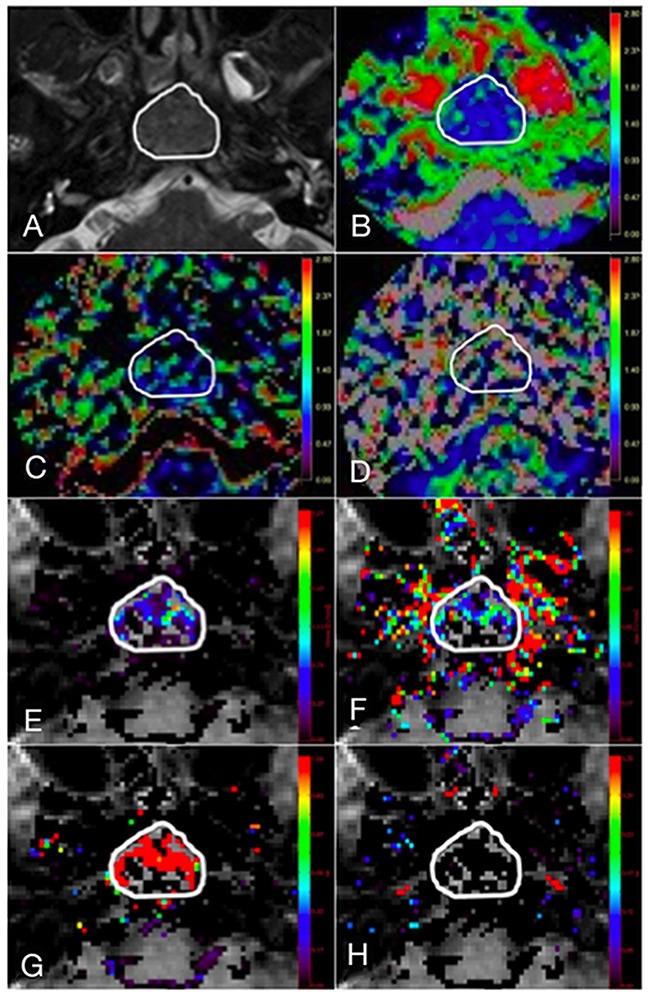
A 45-year-old male with stage T3 NPC **(A)** Axial T2-weighted image showing region of interest (ROI) contouring the margin of the NPC, with subsequent co-registration of the ROI onto corresponding **(B)** diffusion map, *D*; IVIM MR perfusion related maps, **(C)** *f* and **(D)** D*; quantitative DCE MR perfusion maps, **(E)** *K^trans^*, **(F)** *K_ep_*, **(G)** *V_e_* and **(H)** *V_p_*.

Region-of-interest (ROI) was manually drawn by two independent head-and-neck radiologists to contour the border of NPC on each slice of the axial STIR T2-weighted images (Figure 4A), avoiding the inclusion of air and adjacent anatomic structures, such that the total tumor volume was analyzed. Total tumor volume was calculated using segmentation method as described previously [32]. It was then co-registered to the diffusion (Figure 4B) and perfusion (Figure 4C–4H) maps for subsequent analysis using Image J (NIH, Bethesda, MD). Each lesion was measured twice in two separate sessions at 2 weeks apart to ensure reproducibility. The obtained IVIM-MR and DCE-MR parameters for each tumor were calculated on a pixel-by-pixel basis and expressed as mean values of all pixels within the volume of interest.

### Statistical analysis

Patients were stratified into two groups for the evaluation of AJCC stage by combining AJCC stage I and II (*n*=29) into low stage group, and AJCC stage III and IV (*n*=46) into high stage group for statistical analysis.

Normality test was performed using Shapiro-Wilk test, with the DCE and IVIM parameters showing approximately normal distribution (all *P*>0.05). Univariate analysis using Student's t-test was performed to compare IVIM-MR parameters (*D*, *f*, D*) and DCE-MR parameters (*K^trans^*, *K_ep_*, *V_e_* and *V_p_*) between low and high AJCC stages. Multiple logistic regression analysis was performed to gauge their independent predictive values. Receiver operating characteristic (ROC) curves were derived with respective cut-off values determined to accommodate best diagnostic accuracy. Comparison of the accuracy of different ROC curves was carried out by given *p* values. Correlation between IVIM-MR perfusion-related parameters and DCE-MR parameters were determined by using Spearman's rank correlation. All statistical analyses were performed by SPSS version 21 (SPSS Inc, Chicago, IL, USA). Bonferroni correction was performed to account for the multiple comparisons.

## Abbreviations

AIF: arterial input function
AJCC: American Joint Committee on Cancer
AUC: area under curve
*D*: pure diffusion
*D**: pseudodiffusion coefficient
DCE: dynamic contrast enhanced
DW: diffusion weighted
*f*: perfusion fraction
FFE: fast field echo
FOV: field of view
*K^trans^*: volume transfer constant of Gd-DOTA
*K_ep_*: rate constant of Gd-DOTA
IVIM: intravoxel incoherent motion
MR: magnetic resonance
NPC: nasopharyngeal carcinoma
ROC: receiver operating characteristic
ROI: region of interest
SD: standard deviation
SNR: signal-to-noise ratio
SPIR: spectral presaturation inversion recovery
STIR: short T1 inversion recovery
TFE: turbo-field-echo
TR/TE: repetition time/echo time
TSE: turbo-spin-echo
*V_e_*: extracellular volume fraction
*V_p_*: blood volume fraction
